# Correction: Sprinting performance is linked to surface activity in scorpions

**DOI:** 10.1242/jeb.252950

**Published:** 2026-06-23

**Authors:** Eran Gefen, Shoval Atiya, Li-Mor David, Stav Talal

There was an error in *J. Exp. Biol.* (2026) **229**, jeb251978 (doi:10.1242/jeb.251978).

It was incorrectly reported in the Materials and Methods (‘Running assay’ section) and Fig. 1A that running speed was standardized to body size by dividing speed by the scorpion's carapace length. In fact, the running speed data were divided by body length (prosoma+mesosoma). In addition, the number of *S. fuscus* individuals in Fig. 1A was incorrectly reported as 39 – this should be 40.

**Fig. 1A (corrected panel).**
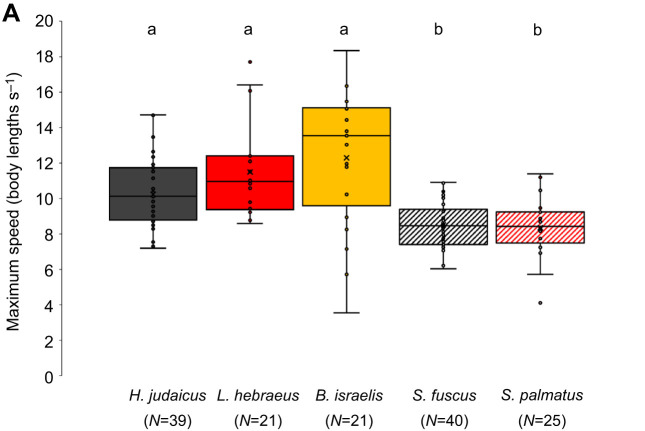
**Running assay results.** Maximum running speed (A) for the five study species: *Hottentotta judaicus*, *Leiurus hebraeus*, *Buthus israelis*, *Scorpio fuscus* and *Scorpio palmatus*. Box plots show median, upper and lower quartiles and 1.5 times the interquartile range; circles are individual data points. Different letters indicate statistically significant differences (Bonferroni-corrected multiple comparisons of means, *P*<0.05).

**Fig. 1A (original panel).**
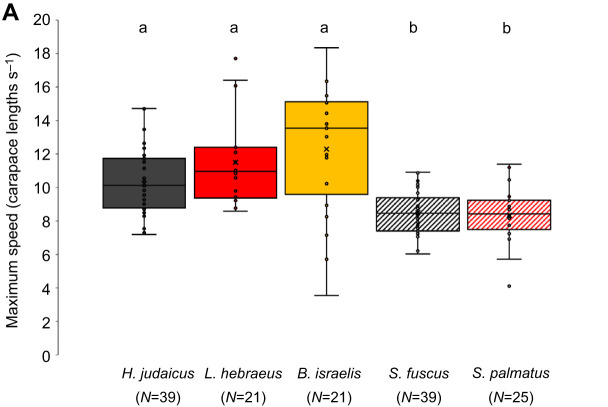
**Running assay results.** Maximum running speed (A) for the five study species: *Hottentotta judaicus*, *Leiurus hebraeus*, *Buthus israelis*, *Scorpio fuscus* and *Scorpio palmatus*. Box plots show median, upper and lower quartiles and 1.5 times the interquartile range; circles are individual data points. Different letters indicate statistically significant differences (Bonferroni-corrected multiple comparisons of means, *P*<0.05).

The corrected and original versions of Fig. 1A are shown below and both the online full-text and PDF versions of the article have been updated.

The authors apologise to readers for this error, which does not impact the presented values or conclusions of the paper.

